# Research on Automatic Error Correction Method in English Writing Based on Deep Neural Network

**DOI:** 10.1155/2022/2709255

**Published:** 2022-03-10

**Authors:** Lanzhi Cheng, Peiyun Ben, Yuchen Qiao

**Affiliations:** ^1^Zhengzhou Railway Vocational & Technical College, Zheng Zhou, Henan 450000, China; ^2^School of Foreign Languages, Chuzhou University, Chuzhou 239000, Anhui, China; ^3^College of Automation Science and Technology, South China University of Technology, Guangzhou 510640, Guangdong, China

## Abstract

As one of the most widely used languages in the world, English plays a vital role in the communication between China and the world. However, grammar learning in English is a difficult and long process for English learners. Especially in English writing, English learners will inevitably make various grammatical writing errors. Therefore, it is extremely important to develop a model for correcting various writing errors in English writing. This can not only be used for automatic inspection and proofreading of English texts but also enable students to achieve the purpose of autonomous practice. This paper constructs an English writing error correction model and applies it to the actual system to realize automatic checking and correction of writing errors in English composition. This paper uses the deep learning model of Seq2Seq_Attention model and transformer model to eliminate deep-level errors. Statistical learning is combined with deep learning and adopted a model integration method. The output of each model is sent to the n-gram language model for scoring, and the highest score is selected as output.

## 1. Introduction

As one of the most widely used languages in the world, English plays a vital role in communication with the world. Now, China has become the country with the most English learners in the world. English test is an important item in the test of students' ability, and English writing ability is often the focus of the test of English proficiency. For students, due to the impact of mother tongue transfer, they often make some grammatical errors, which affect their expression. As we all know, in order to improve the level of English writing, students must do a lot of writing exercises. However, based on the teacher-student ratio, it is difficult for an English teacher to correct a large number of English compositions of each student. In this way, students cannot get timely feedback on their writing, thus failing to achieve the purpose of autonomous learning. Therefore, it is particularly necessary to develop an automatic error correction model for English writing. With the advancement of science and technology and the development of natural language processing technology, the use of computers for automatic error correction of English writing becomes more feasible at this time [1–5].

English writing inspection includes the structure and content analysis of the upper level, as well as the spelling and sentence grammar error detection and correction. At present, there are relatively good commercial tools for word spelling error detection and correction. However, the correction of grammatical errors in students' English writing is a boring and headache problem, and there is no mature solution at present. If the computer can quickly identify writing errors in sentences and give reasonable suggestions for corrections in time, learners will have a better experience in English learning. However, some of the current commercial or open writing error detection and correction tools have relatively ordinary effects. For example, the grammatical error checking function in Microsoft Word cannot detect common grammatical errors such as subject-verb agreement and misuse of prepositions. And the recognition rate of other errors is relatively low. The error detection rate in the automatic checking function of English writing provided by Juku.com in China is also relatively low. It is not very practical in English learning. Therefore, the breakthrough of this problem will greatly promote the application of computer-assisted English learning [6–10].

Common types of English writing grammatical errors include article errors, preposition errors, verb morphological errors, noun singular and plural errors, and subject-predicate coincidence errors. Automatic error correction in English writing is considered an extremely difficult thing, mainly for the following three reasons. (1) Many grammatical errors are context-related. They are composed of correct words. Without context, it is impossible to distinguish which word is wrong in the context. (2) The frequency of each error is relatively low, but the error modes are diverse. (3) There may be many errors in single-day sentences, which increase the difficulty of machine grammatical error correction. Early machine learning methods cannot cover complex and diverse language models, and their accuracy is low, making it difficult to achieve satisfactory results. The automatic error correction task of English writing has also ushered in new development opportunities, which reduces the influence of corpus on the task of English writing error correction to a certain extent [11–15].

Based on the deep neural network, this paper proposes a new automatic error correction model for English writing. The main contributions are as follows: first, the Seq2Seq_Attention baseline model is determined. Then, a Seq2Seq_Attention model based on the subword BPE algorithm is proposed. Next, today's hot Transformer network is used to build an automatic error correction model for English writing and introduce curriculum learning strategies and masked sequence-to-sequence strategies to improve model performance. Besides, the performance of the model is improved based on data processing and data amplification. Finally, the method of model integration is introduced to further improve the performance of the model.

## 2. Related Work

Rule-based methods were widely used in grammar checkers. Errors that were easier to find could be checked with simple rules, such as repeated punctuation. If an English sentence was more complicated, it needed to be checked with more complicated rules. The earlier versions of the rule-based grammar checker included EasyEnglish [16] and Park's grammar checker [17]. The most widely used tools were Microsoft Word, WordPerfect [18], and Grammarian Pro X [19], all of which could be used in multiple languages. They all used certain grammatical rules to check sentences or phrases. Therefore, the main disadvantage was that it is restricted by different languages, and a rule could not be reused by multiple languages. EasyEnglish was an English writing checker developed by IBM. It used the syntax tree represented by the network diagram to check and find errors. It used patterns to formalize writing errors and then matched them with the constructed syntactic tree. This was a writing checker developed based on a rule-based approach and English groove grammar. However, when a sentence could not be parsed correctly and a complete grammar tree could not be constructed, the writing checker would not work well. In addition, whether it was a commercial or free tool, the grammar checker currently had no publicly released version. Park's grammar checker was a writing checker specially developed for English learners who used English as a second language to check typical errors in composition. It was a writing checker implemented by combining the rule method and the Prolog method. The writing checker could manually add rules, and new rules could replace invalid old rules. These rules could give users feedback information but would not return any information for the correct sentence.

Another method that was widely used in grammar checking was the method of statistical data analysis. Due to the rapid development of statistical machine learning methods, corpus linguistics, which used corpus as the research foundation and object, had risen rapidly. This method had been more and more recognized by researchers and had been widely used [20]. Literature [21, 22] proposed a grammatical error correction technology based on the noise channel model, which used the context of the entire sentence to correct the sentence. The basic theory of the noise channel model mainly included two parts, namely the basic language model and the noise model. The former was a probabilistic model, which generated a sentence without errors according to a given probability. The literature [23] used a variety of vocabulary and part-of-speech features, including verb phrases and nouns of adjacent prepositions, part-of-speech tags, word lemmas, to evaluate a large amount of data including newspaper news articles and English articles of intermediate and advanced English students. The error correction range covered 34 commonly used prepositions, and the final accuracy of the maximum entropy algorithm was 69%. Literature [24] used complex grammatical features, WordNet category features, and various types of part-of-speech features. At the same time, some grammatical relations extracted from the syntactic tree were also used. These grammatical relationships could be used as strong preposition checking features. Then, the data subset of the British National Corpus was used as the test set, and the correct rate was 75.6%, which was a relatively good result. Literature [25] proposed a method based on memory learning to select articles. The features he used were extracted from the Pennsylvania sentence database, for example, the part of speech of the head word, the head word of the noun phrase, and other qualifiers in the noun phrase. There were also some features extracted from the translation system from Japanese to English, such as the semantic category of the head word of noun phrases and the tendency of countability. The highest check accuracy rate obtained by the model was 83.6%. Literature [26] used the applied log-linear model to automatically recover the missing articles. It described a competitive classification model to generate and describe the articles, thereby recovering the correct sentence. Literature [27] described a method that uses a maximum entropy classifier to select articles for noun phrases, and some features of the context are used in this process. When the training set size of the classifier reached 6 million noun phrases, the accuracy of the classification method reached 87.99%.

## 3. Method

This chapter first determines the baseline model of Seq2Seq_Attention (S2SA). Then, based on data preprocessing and data amplification, the performance of the baseline model is improved. Then, a Seq2Seq_Attention model based on the BPE subword level is proposed called S2SA-BPE. Then, an English error correction model is built based on Google's Transformer model and introduced curriculum learning strategies and masked sequence-to-sequence training strategies to further improve the correction results. Finally, the N-gram language model and the deep learning model are integrated.

### 3.1. Error Correction Based on S2SA

**Figure 1:**
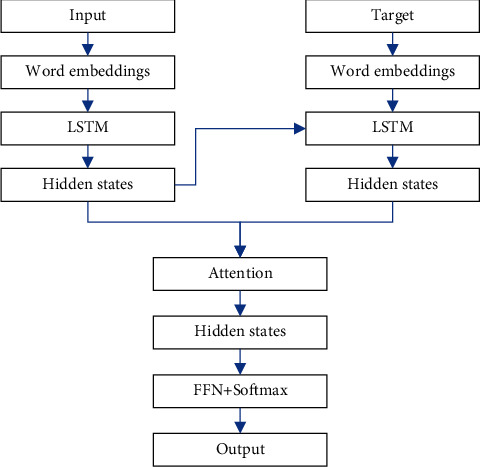
The structure of S2SA.

This paper chooses to build an error correction baseline model based on the S2SA model for the following reasons. (1) S2SA model is a more classic model in neural network translation technology, and its position in neural network translation is equivalent to that of word2vec in the text representation. This model introduces the Attention mechanism, which breaks the restriction that the decoder can only use the encoder to finally fix the vector result. This allows the decoder to focus on the input text that is important for predicting the next target word. In addition, you can also observe the changes in the attention weight matrix to know the source input text corresponding to the target word. This helps to deepen the understanding of the model. (2) The idea of the S2SA model is relatively simple, easy to understand, and its code structure is relatively simple, which will speed up the deployment of the model. Even if the baseline model is not the final model, it can be iterated quickly, thereby reducing unnecessary time costs. (3) The baseline model is easier to deploy, generally consists of relatively few trainable parameters, and can quickly match the data without much processing. The most important thing is to facilitate research; that is, most of the errors encountered can be easier to locate the errors in the data or the defects of the model. (4) The baseline model is conducive to understanding the data, and finding out the errors in the process of constructing the baseline model is very constructive for discovering deviations and specific errors in the data. (5) The baseline model is conducive to understanding the task and helps to understand which part of the project is more difficult and which part is simpler. According to this idea, it is helpful to locate which aspect of the model should be improved, so as to better solve the difficult part. The structure of S2SA is illustrated in Figure 1.

Encoder input is the word embedding, and the output is the hidden layer state.

(1) $$\begin{array}{c} h_{t}=E_{\text{LSTM}}\left(x_{t},h_{t-1}\right), \end{array}$$

where *E*_*LSTM*_ is LSTM encoder.

Decoder input is the word embedding, and the output is the hidden layer state.

(2) $$\begin{array}{c} s_{t}=D_{\text{LSTM}}\left(y_{t},s_{t-1}\right), \end{array}$$

where *D*_*LSTM*_ is LSTM decoder.

The context vector *c*_*i*_ is a weighted average of the hidden layer state.

(3) $$\begin{array}{c} c_{i}=\sum w_{ij}h_{j}. \end{array}$$

The weight for the hidden layer state is calculated as follows:

(4) $$\begin{array}{c} w_{ij}=\frac{\exp\left(e_{ij}\right)}{\sum^{}\text{exp}\left(e_{ik}\right)}. \end{array}$$

The weight with the hidden state is calculated as follows:

(5) $$\begin{array}{c} e_{ij}=\text{score}\left(s_{i},h_{j}\right). \end{array}$$

The context vector, as well as the hidden state, is concatenated as follows:

(6) $$\begin{array}{c} p_{t}=\text{tanh}\left(W_{c}\left[c_{t};s_{t}\right]\right). \end{array}$$

The final output probability is calculated as follows:

(7) $$\begin{array}{c} p=\text{softmax}\left(W_{s}p_{t}\right). \end{array}$$

### 3.2. Error Correction Based on S2SA-BPE

The S2SA model based on word level has the following disadvantages: (1) There are usually many words in the vocabulary that share a vocabulary unit but have different grammatical forms. (2) There are unknown words and rare words. Unknown words refer to words that are not in vocabulary and are marked as OOV words. Rare words refer to some words in the vocabulary that appear too few times in the training corpus so that they cannot be fully trained to obtain good word vectors. (3) There is no perfect word segmentation algorithm in any language. An excellent word segmentation algorithm should be able to divide any sentence into a sequence of lexical units and grammatical forms. Therefore, this paper uses a Seq2Seq_Attention model (S2SA-BPE) based on the BPE subword level, which effectively alleviates the problem of unknown and rare word translation and improves the performance of the model. The S2SA-BPE algorithm implements a text representation unit that is between characters and words and is also different from character n-grams, achieving a more balanced state in terms of vocabulary capacity.

**Figure 2:**
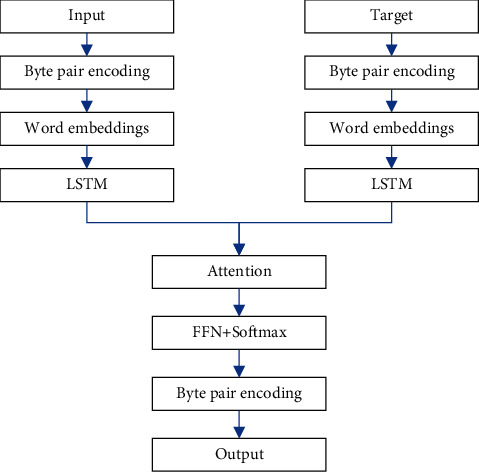
Structure of S2SA-BPE.

Byte pair encoding is a data compression method that uses an unfamiliar byte to replace the byte that frequently appears in the sentence. We use this algorithm to split words and merge characters or character sequences. The steps of the BPE learning algorithm are as follows: (1) the symbol vocabulary is initialized and added all the characters to the symbol vocabulary. Special symbols are added to the end of words. (2) All the symbols are counted, find the most frequent character pair, and replace it with a new character. (3) Each time it is merged, and a new character is generated, which means n-gram character. (4) Common n-grams characters will be merged into one symbol at the end. (5) The final symbol vocabulary size is the sum of the initial size and the number of merge operations. The number of operations is the only hyperparameter of the algorithm. The structure of S2SA-BPE is illustrated in Figure 2.

Existing word segmentation algorithms generally target normal texts and apply standard word segmentation algorithms to the task of correcting English texts. Segmentation of texts with errors will lead to mis-segmentation. Moreover, the word segmentation algorithm itself has the problem of ambiguity segmentation; that is, the word segmentation process will introduce additional error information with a high probability. In addition, the existing translation models based on word level generally limit the size of the vocabulary to alleviate the problem of excessive calculation of the softmax function. The limited vocabulary size will cause rare words to become unknown words, which will damage the performance of the model. Therefore, the S2SA-BPE method can alleviate the above problems to a certain extent.

### 3.3. Error Correction Based on Transformer

This paper builds an English writing error correction model based on the Transformer network because of the following points. (1) In terms of parallel computing capabilities, the current input of the RNN network depends on the input at the previous time, which makes it impossible to parallelize. The Transformer introduces the Attention mechanism to reduce the distance between any two characters in the text to a constant, which helps to alleviate the problem of RNN's long-distance dependence. And due to matrix operations, its parallel computing power is also better than RNN. (2) In terms of computational efficiency, self-attention is more computationally efficient than RNN and CNN. (3) In terms of semantic feature extraction capabilities, Transformer surpasses RNN and CNN. (4) In terms of long-distance feature capture capability, the CNN feature extractor is significantly weaker than the Transformer. (5) In terms of comprehensive feature extraction capabilities represented by machine translation tasks, Transformer has a higher performance than RNN.

Based on the above five factors, this paper chooses to build a Transformer-based error correction model for English writing to further improve the effect of error correction. A big advantage of Transformer is the global receptive field; that is, RNN/CNN can only see part of the context at a time. In Transformer, each node can directly interact with other nodes. But, there are pros and cons, because Transformer does not introduce a strong prior. Therefore, a large amount of data is needed to learn a certain statistical relationship of the data from scratch. Practice has proved that the effect of Transformer on small data sets is not as good as RNN/CNN. But when there is a large amount of training data, Transformer will have a higher upper limit.

### 3.4. Curriculum Learning Strategy

Curriculum learning is similar to the human learning mechanism, that is, learn simple skills first and then learn difficult skills. If the training data are input in a specific order, in other words, the model first learns from simple data and then learns difficult data after the model has a certain ability, which is in line with human intuition. At the same time, from the perspective of machine learning, this method can also avoid falling into a bad local optimal solution prematurely. This can increase the generation speed and speed up the convergence speed and find a better local minimum in the nonconvex training data.

The curriculum learning method is sensitive to hyperparameters, and this paper uses a curriculum learning method with only one adjustable hyperparameter, called competence-based curriculum learning [28].

There are two crucial concepts in the learning strategy method of this course: difficulty and competence. Difficulty represents the difficulty value of a training sample. Its value is determined by the sentence length and the relative word frequency of the word. The calculation is as follows:

(8) $$\begin{array}{c} \text{diff}\left(s_{i}\right)=-\sum_{k=1}^{N}\text{log}\left(f_{k}\right), \end{array}$$

where *s*_*i*_ is the ith sample, *N* is the length of the sample sentence, *f*_*k*_ is the relative word frequency of the kth word in the sample.

Competence is a value between 0 and 1, which represents the progress of model training and is defined as a function of the model state. Specifically, this method defines the model's ability *c*(*t*) at time t as the proportion of training data allowed to be used at time *t*. The training samples are sorted according to their difficulty, and the model only allows their top *c*(*t*) part to be used at time *t*. Linear function and root function are two calculation methods.

(9) $$\begin{array}{c} {\text{comp}}_{L}\left(t\right)=\text{min}\left(1,\frac{\left(1-c_{0}\right)\text{t}}{\text{T}}+c_{0}\right),\\ {\text{comp}}_{R}\left(t\right)=\text{min}\left(1,{\left(\frac{\left(1-c_{0}^{m}\right)\text{t}}{\text{T}}+c_{0}^{m}\right)}^{1/m}\right), \end{array}$$

where *c*_0_ is the initial value, *T* is the time step threshold. When the threshold is exceeded, the model is considered to be fully capable, and *t* is the time step.

**Figure 3:**
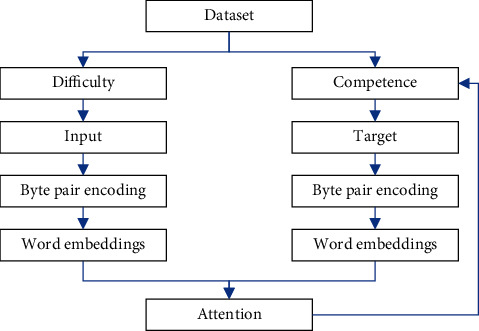
Based on the curriculum learning model frame diagram.

In this paper, the curriculum learning strategy is applied to the English writing error correction task to achieve the purpose of improving performance. The model is illustrated in Figure 3.

The competence of this paper is in addition to the linear form and root form mentioned above. A method for selecting training data based on loss is also proposed. The most intuitive reflection of the strength of the model is the loss of the model. The calculation formula is as follows:

(10) $$\begin{array}{c} {\text{comp}}_{\text{Loss}}\left(t\right)=\text{min}\left(1,{\left(\frac{1-\text{loss}}{\text{T}+c_{0}}\right)}^{1/2}\right). \end{array}$$

### 3.5. Masked Sequence-to-Sequence Strategy

**Figure 4:**
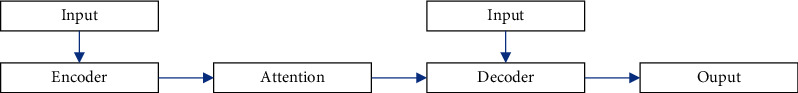
MASS method architecture diagram.

Most of the existing pretraining models are based on natural language understanding tasks and have achieved excellent results, which have attracted more and more attention. However, in sequence-to-sequence natural language generation tasks, such as machine translation, summary generation, automatic question and answer, few pretrained models are targeted for such tasks. Literature [29] proposed a pretraining method for natural language generation tasks: masked sequence-to-sequence pretraining (MASS). This method uses the encoder-decoder framework to reconstruct a sentence segment: its encoder randomly masks multiple consecutive features of the input sentence. Then, the decoder tries to predict the features that are concealed, and its model architecture is shown in Figure 4.

MASS pretrains the encoder and decoder jointly in two steps. (1) By predicting the feature of the sentence that is masked in the encoder, MASS forces the encoder to understand the meaning of the feature of the unmasked sentence. (2) By masking the decoder input that is not masked at the source, MASS forces the decoder to rely more on the representation of the source. So as to better promote the union between encoders.

The MASS method has the following advantages: (1) The decoder side masks all words to facilitate the decoder to extract more information to improve the prediction results, thereby facilitating joint training. (2) In order to provide more information to the decoder, the encoder is forced to extract the information of the unmasked words, thereby improving the ability of the encoder to extract information. (3) Decoder is used to predict the continuous words that are obscured, which can promote the language modeling ability of the decoder. In order to improve the effect of the error correction model, this paper introduces the MASS pretraining method to the error correction task for the first time. This paper generates masking data based on char and word. It should be noted that the features are masked sequentially, not randomly.

### 3.6. Data Preprocessing and Data Amplification

The reasons for data preprocessing and data amplification in this paper are as follows: (1) The higher the quality of the training data, the better the model performance. The quality of the data determines the upper limit of task performance, and necessary data preprocessing techniques can be used to approach the upper limit of task performance. (2) The more the training data, the better the model performance. Data are the core driving force of neural network models. Massive training data are one of the important reasons for the success of neural network models.

Processing the data is essential while developing a deep learning model in order to lessen the data's complication. A suitable model is established, which will help the model fit the data better and increase the pace at which it converges and improves the model's effectiveness.

Therefore, based on the above viewpoint, this paper uses edit distance to process the corpus. Edit distance [30] is also called Levenshtein distance, which is a tool to measure the similarity of two texts. Its specific meaning refers to the minimum number of editing operations required to convert one text to another. The editing here generally includes three types: inserting characters, deleting characters, and replacing characters.

In the field of natural language processing, data amplification is generally achieved in three ways. (1) Direct method searches for data related to the task and directly performs data amplification. (2) The indirect method, through the pretraining model, directly fine-tunes the trained model based on the data of its own task. This method generally requires higher hardware. (3) The method of data modification, that is, the four simple operations of synonym substitution, random insertion, random exchange, and random deletion, is used to achieve data amplification. But in the field of natural language processing, this method is rarely used, because simple addition, deletion, and modification operations can easily damage the performance of the model. In the data amplification experiment in this chapter, the direct amplification method is used to combine different corpora.

### 3.7. Model Integration

Model integration is to build a series of models and uses a certain strategy to combine the built models. Then, a model with higher accuracy, better stability, and generalization effect can be obtained. At present, model integration has become a weapon in various competitions or tasks. There are many existing integration methods, but the most classic methods are Bagging and Stacking. Many methods have also been developed from this. The main idea of Bagging is to randomly select a part of the sample from the overall sample with a replacement for training and obtain multiple models by repeating the operation multiple times. Then, the average is voted or taken as the resulting output. Stacking is a layered model integration method. The input of the first layer is the original training set, and the second layer uses the output of the first layer model as the training set for training.

**Figure 5:**
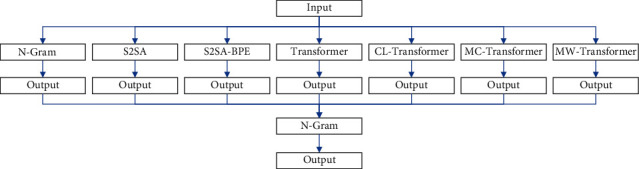
Multimodel integration framework diagram.

The model ensemble method of this paper is to learn from the Bagging method. N-gram, S2SA, S2SA-BPE, Transformer, Curriculum learning strategy-based Transformer (CL-Transformer), char-based masked sequence-to-sequence strategy Transformer (MC-Transformer), and word-based masked sequence-to-sequence strategy Transformer (MW-Transformer) is combined for integration. The output results are scored using the N-gram language model. The highest score is used as the final output, and its multimodel integration framework is shown in Figure 5.

## 4. Experiment and Discussion

### 4.1. Experimental Environment

The models used in this chapter are all end-to-end learning networks, and a suitable experimental environment is built according to the characteristics of the end-to-end learning network. This paper is based on the Python language and uses the PyTorch framework to code the model. The experimental environment is shown in Table 1.

### 4.2. Dataset and Metric

The training forecast in this article is mainly divided into two parts. The first part comes from the International Corpus Network of Asian Learners of English (ICNALE), and the second part comes from the Brown Corpus. The testing is expected to come mainly from the learner corpus.

The *F* value evaluation is commonly used internationally to evaluate the effect of grammar checking. *F* value evaluation is also a commonly used evaluation standard in the field of natural language processing, and its calculation formula is

(11) $$\begin{array}{c} F_{\beta }=\frac{\left(1+\beta^{2}\right)PR}{\beta^{2}P+R}, \end{array}$$

where *β* is the parameter, *P* is precision, and *R* is recall rate. The calculation formula of *P* and *R* is as follows:

(12) $$\begin{array}{c} P=\frac{A}{A+B},\\ R=\frac{A}{A+C}, \end{array}$$

where *A* represents the number of sentences that actually contain grammatical errors among the sentences marked by the model as containing grammatical errors. *B* represents the number of sentences that do not contain grammatical errors among the sentences marked by the model as containing grammatical errors. *C* represents the number of sentences with grammatical errors that are not marked by the model.

This article uses *F*_0.5_ instead of *F*_1_, because in practical applications, we focus more on accuracy. In *F*_0.5_, it is emphasized that the accuracy rate is twice the recall rate, and in *F*_1_, the accuracy rate and the recall rate are equally important.

### 4.3. Evaluation on S2SA

**Figure 6:**
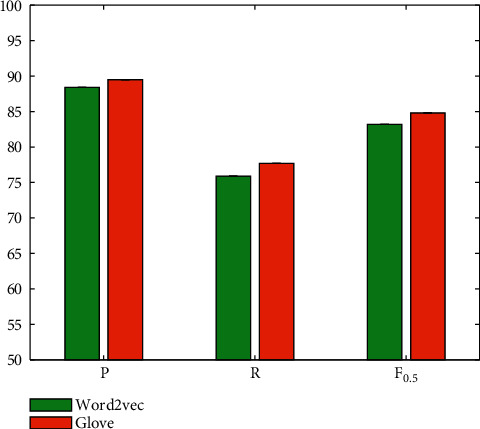
Evaluation on S2SA for different word vectors.

S2SA is the baseline model of this paper. It uses word vectors to represent the input text. Both encoder and decoder use a two-layer two-way LSTM network. In order to obtain better text representation, two kinds of word vectors are used, namely Word2vec word vector and GloVe word vector. The result is illustrated in Figure 6.

The data results show that the performance of the baseline model based on the Word2vec word vector is slightly lower than that of the baseline model based on the GloVe word vector. The latter achieves 1.1%, 1.8%, and 1.6% performance improvements on three performance indicators. In subsequent experiments, GloVe word vectors are used.

### 4.4. Evaluation on S2SA-BPE

**Figure 7:**
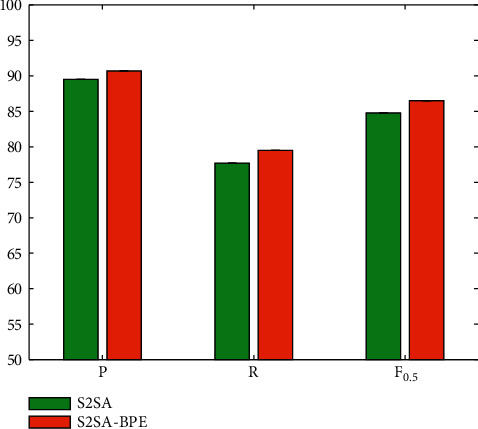
Evaluation on S2SA-BPE.

To solve the problem of unknown words and rare words that often appear in the word level model, BPE subword technology is introduced. The word segmentation tool used is a trained BPE subword model, and the remaining model parameters are consistent with the baseline model settings. In order to verify the effectiveness of this model, S2SA-BPE and S2SA are compared, and the experimental results are shown in Figure 7.

The data results show that under the same data and the same parameters, the performance of the model based on S2SA-BPE surpasses the performance of the model based on word level. The improvements in the three performance indicators are 1.2%, 1.8%, and 1.7%. The subword strategy can improve the performance.

### 4.5. Evaluation on Curriculum Learning Strategy

**Figure 8:**
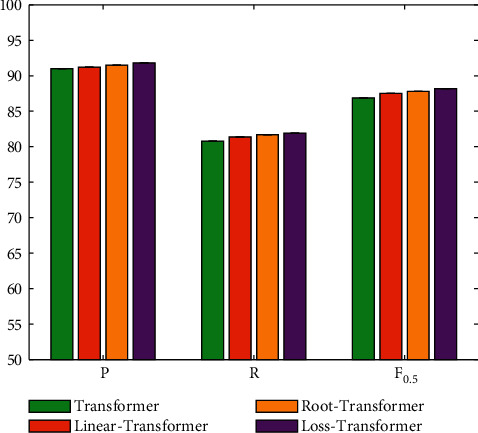
Evaluation on curriculum learning strategy.

The curriculum learning strategy imitates the human learning mechanism, that is, learn simple knowledge first, and then learn difficult knowledge. This learning method can reduce the probability of falling into a local optimal solution and speed up the convergence speed of the model. The input of the model is that the difficulty value of the input text is less than the current ability value of the model, and three experiments are set up according to the different calculation methods of competence. They are the linear method, root method, and loss method, and the rest are consistent with the Transformer model parameters. The result is illustrated in Figure 8.

It can be seen that curriculum learning strategies can effectively improve the performance of error correction in English writing. It should be noted that compared with the traditional Transformer method, no matter which strategy can obtain performance improvement. But compared with the linear and root methods, the loss method designed in this paper can achieve the best performance improvement. This proves the effectiveness of this design in this paper.

### 4.6. Evaluation on Masked Sequence-to-Sequence model

**Figure 9:**
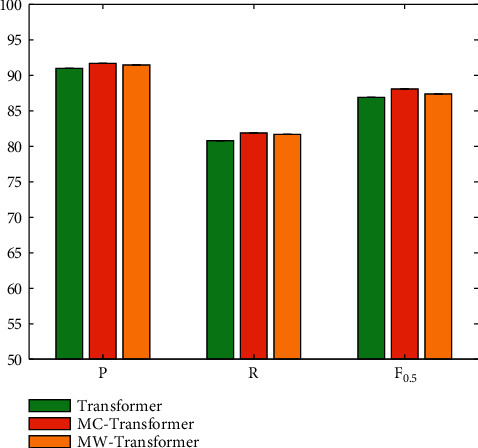
Evaluation on masked sequence-to-sequence strategy.

The pretraining method is used to indirectly increase the size of the data set. The encoder and decoder are jointly trained by masking the sequence-to-sequence model, breaking the limitation that the pretraining model can only train a certain part. The model first uses the preprocessed corpus for pretraining, and the pretraining part can be divided into two parts. One is a model based on word level, and the other is a model based on word level. In order to compare these two different methods, this paper conducts a comparative experiment, and the results are shown in Figure 9.

It can be seen that the MASS strategy can effectively improve the performance of error correction in English writing. It should be noted that compared with the traditional Transformer method, no matter which strategy can obtain performance improvement. But compared with the word method, the char method designed in this paper can achieve the best performance improvement, which can achieve 0.7%, 1.1%, and 1.2% gains on precision, recall, and *F*_0.5_.

### 4.7. Evaluation on Data Preprocessing and Data Amplification

**Figure 10:**
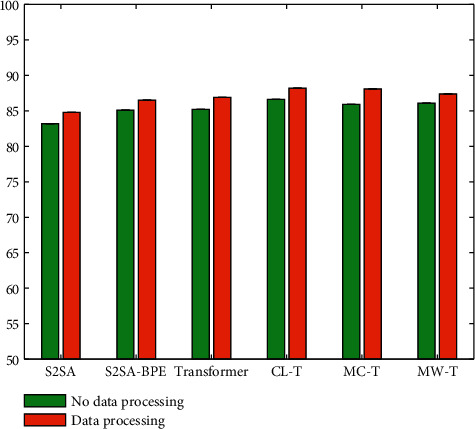
Evaluation on data preprocessing and data amplification.

In this paper, data preprocessing and data amplification are carried out on the data used in the experiment. In order to verify the effectiveness of this data processing method, this paper conducts a comparative experiment. The performance of each model with data processing and without data processing is compared, and the experimental results are shown in Figure 10. CL-T is CL-Transformer. MC-T is MC-Transformer. MW-T is MW-Transformer.

It can be seen that after using data preprocessing and data amplification methods on each individual model, the error correction performance of the model will be improved accordingly. This experiment proves the effectiveness of the data processing method in this paper.

### 4.8. Evaluation on Multimodel Aggregation

The writing error correction model designed in this article is an aggregation model, which is aggregated from S2SA, S2SA-BPE, Transformer, CL-Transformer, MC-Transformer, and MW-Transformer. In order to verify the effectiveness of this aggregation model, this paper compares the separate model with the aggregated model. The results are shown in Table 2.

The data show that the aggregation model can achieve the best performance compared to each individual model. This proves the reliability and correctness of the error correction algorithm for English writing based on the aggregation model proposed in this paper.

### 4.9. Comparison with Other Methods

In order to further verify the effectiveness of the method in this paper, the aggregation model is compared with other English writing error correction methods. The compared methods in this work include the naive Bayesian model (NBM), decision tree model (DTM), maximum entropy model (MEM), and KNN. The result is illustrated in Table 3.

Compared with other error correction methods, the aggregation model method designed in this paper can obtain the best performance. Compared with the best KNN method listed, the three performance indicators have been improved by 3.2%, 2.2%, and 3.7%, respectively.

## 5. Conclusion

With the development of artificial intelligence and computer science and technology, natural language processing technology has developed rapidly, providing a theoretical and technical basis for the intelligent assistance of English. A large number of teaching and tutoring software has emerged in the following. As an important part of English learning, writing has become a difficult challenge for learners in the learning process. For students, the strength of the ability to use English writing directly affects the level of English proficiency. At the same time, this will also affect English reading and speaking skills. Based on the deep neural network, this article is dedicated to developing an intelligent model for correcting various errors in English writing. This can not only be used for automatic inspection and proofreading of English writing but also enable students to achieve the purpose of autonomous practice. First of all, this paper determines the S2SA baseline model. Then, an S2SA-BPE model based on the subword BPE algorithm is proposed. Afterward, we used the now hot Transformer network to build an error correction model for English writing. And the curriculum learning strategy and the masking sequence-to-sequence strategy are introduced to improve the performance of the model. Then, the model performance based on data processing and data amplification is improved. Finally, the method of model integration is introduced to efficiently aggregate the various submodels designed. This can further improve model performance.

## Figures and Tables

**Table 1:** The experimental environment.

Item	Type
CPU	Intel core i7-8700K
GPU	NVIDIA GeForce RTX 3090ti
Operating system	Ubuntu 20.04
Deep learning framework	Pytorch 1.8

**Table 2:** Evaluation on multimodel aggregation.

Model	*P*	*R*	*F*_0.5_
S2SA	89.5	77.7	84.8
S2SA-BPE	90.7	79.5	86.5
Transformer	91.0	80.8	86.9
CL-transformer	91.8	81.9	88.2
MC-transformer	91.7	81.9	88.1
MW-transformer	91.5	81.7	87.4
Ours	92.5	84.3	89.5

**Table 3:** Comparison with other methods.

Method	*P*	*R*	*F*_0.5_
NBM	79.8	68.1	73.5
DTM	84.3	72.5	77.8
MEM	87.5	81.4	84.3
KNN	89.3	82.1	85.8
Ours	92.5	84.3	89.5

## Data Availability

The datasets used are available from the corresponding author on reasonable request.
